# Robot-Assisted Urological Oncology Procedures, Outcomes, and Safety in Frail Patients: A Narrative Review of Available Studies

**DOI:** 10.5152/tud.2024.23198

**Published:** 2024-01-01

**Authors:** Nikolaos Kostakopoulos, Themistoklis Bellos, Evangelos Malovrouvas, Stamatios Katsimperis, Athanasios Kostakopoulos

**Affiliations:** 1 Department of Urology, Metropolitan General, Athens, Greece; 2 Department of Urology, Aberdeen Royal Infirmary, Aberdeen Urology Unit, NHS Grampian, United Kingdom; 3 Department of Urology, University of Athens, Sismanogleio General Hospital, Athens, Greece

**Keywords:** Frailty, robotic urological procedures, robotic prostatectomy, robotic cystectomy, robotic nephrectomy

## Abstract

In this study, we assess the impact of frailty on the success rate and risk of complications of robot-assisted urological procedures and introduce effective preoperative screening tools to evaluate frail patients’ fitness to tolerate robot-assisted urological surgery. We performed a search of electronic databases for available studies, published up to August 2023, investigating the outcomes of robot-assisted urological oncology procedures and their safety in frail patients. Sixteen studies were ultimately selected, investigating the implications of frailty in robot-assisted radical cystectomy, robot-assisted partial nephrectomy, and robot-assisted radical prostatectomy. All the studies used the Clavien–Dindo classification of complications with serious complications considered as Clavien–Dindo ≥3. Frail patients significantly benefit from robot-assisted urological procedures in comparison to open surgery, with lower rates of blood transfusion and a shorter length of stay. However, they also have a higher risk of postoperative complications than non-frail patients, as well as increased rates of conversion to open, total hospital costs, and in-hospital mortality after robot-assisted procedures. Robot-assisted urological procedures can improve the postoperative recovery of frail patients in comparison to open surgery. Reliable frailty indexes such as the Johns Hopkins indicator and simplified frailty index, as well as the Geriatric 8 screening tool, should be routinely used in the preoperative assessment of frail patients to optimize surgical decision-making.

**Main Points**

Higher rates of robot-assisted urological oncology procedures, in the population of elderly and frail patients are performed in the last decade.
We investigate the safety of robotically-assisted urological procedures in frail patients and their outcomes in comparison to open surgery.
The aim of this review was to compare and suggest the most effective preoperative screening tools to assess these patients’ fitness to tolerate minimal invasive urological surgery.

## Introduction

The progressive increase in life expectancy in developed countries has been associated with higher rates of urological malignancies in elderly patients. It is well established that old age is a risk factor for worse recovery after surgery. However, in recent years, frailty has emerged as a more accurate indicator of patients’ health and the outcome of invasive procedures. Frailty is a syndrome that includes multiple factors such as a decline in physical strength, endurance, mobility, and loss of weight, mainly affecting the geriatric population. It is associated with a higher risk of complications after oncological procedures and all-cause mortality.^1,2^

In the last decade, the well-established benefits of robot-assisted urological procedures, such as robot-assisted radical cystectomy (RARC), robot-assisted partial nephrectomy (RPN) and robot-assisted radical prostatectomy (RARP), have led to higher rates of these procedures in the population of elderly patients and patients with multiple comorbidities.^1-6^

Besides, the benefits of RARC and intracorporeal urinary diversion concerning the need for blood transfusion and almost all health-related quality of life domains in comparison to open surgery have been proven recently with high-quality randomized control trials.^7,8^

In this review article, we investigate the safety and outcomes of robotic-assisted urological procedures in frail patients and demonstrate the most effective preoperative screening tools to assess these patients’ fitness to tolerate minimally invasive urological surgery.

## Material and Methods

**Figure 1.**
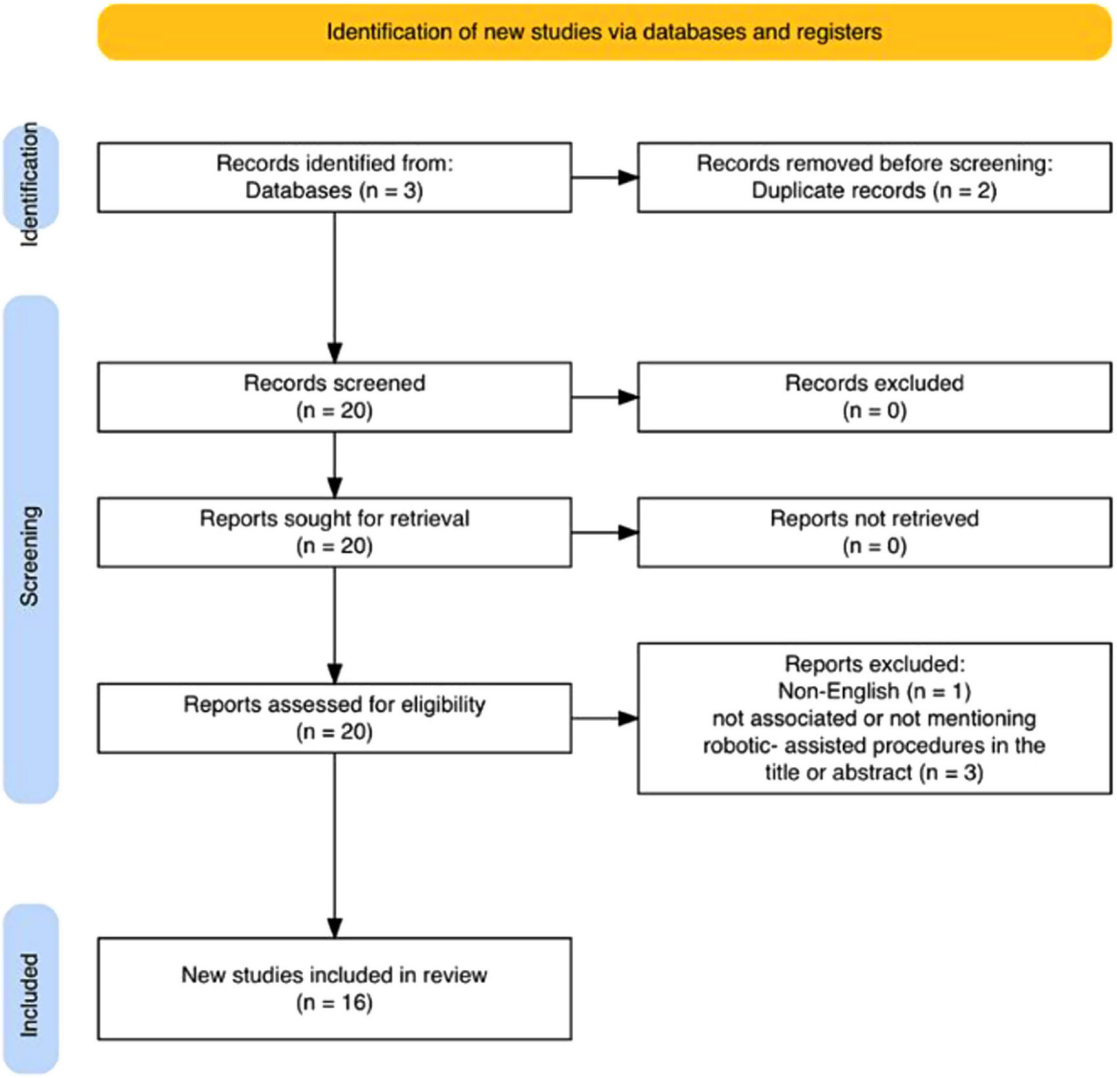
Preferred Reporting Items for Systematic Reviews and Meta-Analyses Flow Diagram.

We searched the available literature, using the MEDLINE (via PubMed), Web of Science, and the Cochrane Library databases for studies published up to August 2023, investigating the impact of frailty on the outcomes and risk of complications of robot-assisted urological procedures, as well as identifying the most accurate frailty indexes for the preoperative assessment of patients, using suitable keywords: “robotic urological procedures,” “frailty,” “robotic prostatectomy,” “robotic cystectomy,” and “robotic nephrectomy,” following Preferred Reporting Items for Systematic Reviews and Meta-Analyses guidelines (Figure 1). A total of 22 studies were identified from the initial search. After title and abstract screening (2 duplicates, 1 non-English, 3 not associated or not mentioning robotic-assisted procedures in the title or abstract), 16 studies were included in the review. Original studies or reviews investigating the association of frailty and robotically-assisted urological oncology procedures were included, either in comparison to laparoscopic and open surgery or to non-frail patients. Case reports or studies that did not include robotically-assisted procedures in the frail were excluded from our review. A non-systematic narrative review was performed.

## Results

Most studies included patients who underwent robotic radical prostatectomy (12 studies), with only 4 studies investigating the association of frailty with the outcomes of robotic partial nephrectomy (RPN) and robotic radical cystectomy (2 studies each). No studies to assess the correlation between frailty and robotic radical nephrectomy, robotic partial cystectomy, or robotic radical nephroureterectomy were found (Table 1).

All the available studies used the Clavien–Dindo classification of complications, with serious complications considered as those with a score ≥3. Most studies included in the literature review were systematic reviews with or without meta-analysis and narrative reviews. Also, case-control studies and retrospective studies were retrieved from our search, and only 1 prospective study. No randomized control trials were identified.

The available studies show that frail patients significantly benefit from robot-assisted urological oncological procedures in comparison to open surgery. Nevertheless, frailty is associated with a higher risk of postoperative complications and worse outcomes than for non-frail individuals and with higher costs for the health care system.

### Robot-Assisted Radical Cystectomy and Frailty

Considered to be the most severe oncological procedure in urology, RARC is associated with the highest morbidity and mortality rates of all other robotic urological procedures. Hence, it is of utmost importance to investigate the procedure’s safety in the population of frail individuals. The percentage of frail patients undergoing RARC in the available studies was 14% (range 9%-18%), while the non-frail RARC percentage was 17% (8%-24%).^1,2^ Frail patients undergoing RARC benefited from a shorter length of stay (LOS) (median 8 vs. 9 days, *P* < .001), in comparison to those having open surgery. Regardless, frailty was significantly associated with a higher risk of postoperative complications in comparison to non-frail patients, as well as higher chance of Intensive care unit admittance.

Total costs were also significantly higher among frail RARC patients, with frailty being a more important predictor of additional costs than the Charlson Comorbidity Index. Most importantly, frail patients were found to have 2 times higher in-hospital mortality than non-frail patients (3% vs. 1.5%, *P* < .05). The most accurate indexes to assess the risk of postoperative complications in frail patients undergoing robotic radical cystectomy were found to be the Johns Hopkins indicator (JHI) and the simplified frailty index (sFI). However, the most commonly used index was the modified frailty index, a reduced 11-item index of the Canadian Study of Health and Aging Frailty Index (CSHA-FI).^1,2^

### Robot-Assisted Partial Nephrectomy and Frailty

In comparison to open surgery, RPN is associated with several advantages for frail patients. In total, RPN, which is the chosen technique in 13% to 40.4% of the patients, has shown lower overall risk for postoperative complications in the frail population (35.3% vs. 48.3%), major complications Clavien–Dindo ≥3 (12.4% vs. 20.4%), as well as lower rates of blood transfusion and shorter LOS, but also increased total hospital costs (*P* < .001).^3,4^

Nevertheless, when compared to the non-frail population, frailty has been found to be significantly associated with a higher rate of complications after RPN. Furthermore, frail patients had a higher likelihood of manifesting postoperative acute kidney insufficiency, since their renal function permanently decreased over time, without improvement during the follow-up period as seen with the non-frail. In addition to this, frailty was implicated with higher rates of other-cause mortality [hazard ratio: 1.67, 95% CI, 1.05-2.66; *P* = .02], although cancer-specific mortality rates did not differ (*P* = .3). In other words, the risk of death from other causes is much higher than the mortality from renal cell carcinoma in the frail population. Thus, frail patients should be carefully evaluated and consulted about the risks and benefits of RPN before choosing to proceed to minimal invasive treatment.^4^

### Robot-Assisted Radical Prostatectomy and Frailty

The most studied robotic urological procedure on frail individuals is robotic radical prostatectomy, and the most widely used frailty index to evaluate these patients is the Geriatric 8 (G8) screening tool.^5^

It is well established that comorbidities such as cerebro-cardiovascular disease or chronic respiratory disease and frailty indexes like the G8 <14 are significant contra-indicators for offering surgical treatment with RARP.^5^ In addition to this, frailty is a proven cause of conversion to open during minimally invasive radical prostatectomy (laparoscopic or robotic), although it does not seem to affect the postoperative quality of life of the patients.^6,9^

Furthermore, it is suggested that frailty and older age (>75) do not affect the oncological outcomes and patient reported outcomes, such as return to continence for patients undergoing RARP, with the exception of erectile function which is negatively affected by senior age.^9,10,11^ However, frailty is associated with an increased risk of postoperative complications, especially severe complications (Clavien–Dindo >IV) and 30-day mortality after RARP, as well as higher rates of moderate-to-severe postoperative pain.^12,13,14^

The increased experience of the surgical community in performing robotic procedures during the last decade has subsequently increased the number of frail individuals who undergo RARP for prostate cancer.^15^ This tendency to perform RARP in more frail patients also derives from indications of high rates of misclassification of these patients between clinical vs. pathological PCa burden.^16^

Regardless of the cause for more frail men undergoing RARP, it is well proven that these patients are also at a higher risk of experiencing postoperative complications, with the rates not being different between open and RARP.^17^

Hence, it is of utmost importance that frail patients are carefully assessed on their fitness to tolerate surgery. The Vulnerable Elders Survey-13 (VES-13) and G8 are accurate and easy-to-use geriatric screening tools that can successfully determine the surgical fitness of frail patients and could potentially substitute life expectancy as the main criterion for choosing RARP as the preferred treatment option.^18^

## Discussion

It has recently been proven that frailty is an important parameter that affects the surgical outcome of major oncological procedures in urology.^19^ The most recent guidelines of the European Association of Urology for urogenital malignancies, such as for prostate cancer, kidney cancer, and muscle-invasive bladder cancer, recommend a preoperative patient assessment concerning their fitness to tolerate oncological procedures, such as radical prostatectomy, partial nephrectomy, and radical cystectomy respectively.^20,21,22^

Less than a decade ago, the first studies investigating the association of minimally invasive urological surgery with frailty were published.^12^ However, the vast majority of evidence for our review is derived from studies from the last 3 years.^1-11,14-18^ As a consequence, no previous reviews are available to summarize the evidence concerning the outcomes of the most common robot-assisted urological procedures in frail patients, although these minimally invasive oncological operations have increased during the last decade.

In this review article, we present for the first time in the literature, to the best of our knowledge, the outcomes and safety of the major oncological robot-assisted urological procedures in frail patients, as well as the most accurate frailty indexes for the preoperative patient assessment.

A study by Rosiello et al^3^ showed that robotic partial nephrectomies in patients with frailty have massively increased from 17% in 2008 to 55% in 2015, with a trend for higher rates in the near future.

Moreover, RPN patients exhibit lower rates of postoperative complications, blood transfusions, and shorter LOS than with open partial nephrectomy. On the contrary, RPN was associated with higher total hospital costs ($50 060 RPN [IQR: $33 369-77 897] vs $39,644 OPN [IQR: $27,093-$60,655]). Nonetheless, Rosiello et al^4^ proved that frail patients are at an increased risk of complications after partial nephrectomy (open or robotic), non-reversible acute kidney injury, and other-cause mortality.

Besides, Abou Heidar et al^15^ showed that RARPs have also significantly increased in frail and comorbid patients from 2011 to 2019.^15^ More specifically, patients with 5-item frailty index ≥2 showed an increase from 9.4% in 2011 to 12.5% in the year 2019 (*P* < .001), while patients with metabolic syndrome index = 3 also showed a rise from 4.1% in 2011 to 6.1% in 2019 (*P* < .001). In addition, patients with an American Society of Anesthesiologists’ score ≥3 also showed an increase from 32.8% in 2011 to 42.4% in 2019 (*P* < .001). Another study showed that 36% of RARP patients had a G8 score ≤14.^23^

This increase in frail patients undergoing RARP did not result in higher rates of major morbidity or mortality, according to the authors, although this could be explained in part by the added experience of the surgeons.^15^ Nevertheless, a recent meta-analysis showed that frailty is associated with a higher risk of severe postoperative complications (≥Clavien–Dindo IV) and all-cause mortality, regardless of the approach being open or robot-assisted.^17^

In a population-based retrospective study by Palumbo et al^2^ it was shown that, as with RARP and RPN, RARC has been offered to a higher rate of frail patients (estimated annual percentage changes +27.1%, *P* < .001), from 0.2% in 2008 to 7.89% in 2015. Frail patients who underwent RARC mainly benefited from a shorter LOS (LOS 8 vs. 9 days, *P* < .001). Apart from this, RARC was associated with higher costs among both frail and non-frail.

Several frailty indexes have been used to preoperatively assess RARC patients; however, a systematic review by Ornaghi et al^1^ found that the JHI and the sFI, an easier-to-use 5-item index based on the CSHA-FI, are the most reliable for identifying patients at higher risk of experiencing postoperative complications.^1^

Similarly, the G8 screening tool is one of the most commonly used frailty indexes for patients undergoing RARP.^5,16,18,21^ The G8 scores range from 0 to 17 and the most used cutoff for frailty is ≤14, with a 65.2% sensitivity and 95.7% specificity for detecting vulnerable prostate cancer patients.^24^ Alternatively, the VES-13 is an easy-to-use screening tool that can predict mortality in patients with prostate cancer.^25,26^ In patients who received androgen deprivation treatment, the sensitivity and specificity of VES-13 in predicting adverse events were 72.7% and 85.7% respectively,^27^ when compared to the Comprehensive Geriatric Assessment, which is the gold standard for assessing the health status of patients but is time-consuming and requires the necessity for several types of experienced physicians, such as geriatrists, urologists, and physical therapists.^18^

Limitations of this review article include its non-systematic design and the small number of available studies, most of which were retrospective or observational. Prospective studies with a larger number of patients will standardize frailty indexes and improve decision-making for robotic surgery, for both the multidisciplinary team of physicians and the patients’ family.

In conclusion, most studies are in agreement that although robot-assisted procedures improve outcomes for frail patients and reduce perioperative morbidity and mortality, frailty is still a significant risk factor that leads to more severe complications than in the non-frail population.

## Conclusion

Frail patients can benefit from robot-assisted urological procedures, but are more susceptible to worse postoperative outcomes and a higher risk of severe complications than the non-frail. As a result, routine preoperative frailty assessment is of utmost importance, using standardized indexes such as the JHI and sFI for RARC and RPN and the G8 screening tool for RARP, in order to safely choose patients who are fit for minimally invasive surgery.

## Figures and Tables

**Table 1.** Studies About Robot-Assisted Urological Procedures in the Frail.^1-6,9-18^

Study/Authors	Type of Robot-Assisted Procedure (%/patients)	Outcomes/Conclusion	Frailty Indexes
1. Ornaghi et al 2020	RARC (&ORC, LRC) (9%-18% of frail patients undergoing RARC or LRC)	Frailty predictive of increased risk of early postoperative major complications, non-home discharge, longer LOS, higher costs, and early mortality.	Johns Hopkins indicator (JHI), 5-item simplified Frailty Index (sFI), 11-item modified Frailty Index (mFI)
2. Palumbo et al 2020	RARC (14% 488/3477 of frail) vs ORC	RARC one-day advantage in LOS	JHI
3. Rosiello et al 2021	RPN (40.4%) vs OPN	RPN lower rates of short-term postoperative complications, blood transfusions, and non-home-based discharge compared to OPN. Additionally, RPN had a shorter LOS than OPN. However, RPN was associated with higher costs.	JHI
4. Rosiello et al 2023	RPN (13%) vs LPN (9.6%) vs OPN (76%)	Frailty higher risk of adverse surgical outcomes and acute kidney injury (AKI) after PN	mFI
5. Kodama et al 2021	RARP (256) vs radiotherapy (RT) non-frail (60) vs RT frail (163)	G8 score and comorbidities have a significant effect on surgical contraindication in patients with localized CaP.	Geriatric 8 screening tool (G8)
6. Luzzago et al 2020	RARP & LRP (57 078)	0.6% conversion to open (strongly associated with patient obesity, frailty, CCI ≥2)	JHI
7. Togashi et al Mar 2021	RARP (41/118 frail)	Frailty not associated with worsening of HRQOL, LUTS, and pad-free continence rates in patients treated with RARP	G8
8. Togashi et al Oct 2021	RARP (74/752 ≥75 years)	Oncologic outcomes and PROs in select patients with prostate cancer aged ≥75 years were feasible and acceptable with RARP.	≥75 years
9. Leyh-Bannurah et al 2022	RARP (669/8937 ≥75 years)	Apart from erectile dysfunction, there was no significant effect on urinary continence recovery, biochemical recurrence- or metastatic progression-free rates after RARP	≥75 years
10. Revenig et al 2014	RARP (15%, also 2.5% RARC and other minimally invasive procedures)	Frail at increased risk of postoperative complications compared with non-frail	Fried criteria
11. Levy I et al 2017	RARP (23 104)	mFI and ASA can predict 30-day mortality for RARP patients better than mFI or ASA alone.	mFI
12. Momota M et al 2020	RARP (61/154, G8 ≤14)	Frailty associated with moderate to severe pain after RARP, with G8<14 NOT sFI.	G8, sFI
13. Abou Heidar NF et al 2023	RARP (66 683)	RARP performed on more frail patients, with no added morbidity or mortality.	5 item frailty index (5-iFI), ASA, Metabolic syndrome index
14. Liakos N et al 2022	RARP (13 765)	Every second senior patient has a misclassification in (i.e., any up or downgrade), and each 4.5th senior has an upgrade in final pathology that translates to an unfavorable PCa prognosis	≥75 years
15 Liu X et al 2022	RARP & ORP (40 518 (23.6%) frail/171 929)	Frailty predictor of severe postoperative complications and all-cause mortality of patients with PCa after radical prostatectomy.	G8, JHI, 5-iFI
16.Yamada Y et al 2022	RARP	Frailty screening tools find unfit patients for surgery preoperatively.	G8, Vulnerable Elders Survey-13 (VES-13)

ASA, American Society of Anesthesiology; CCI, Carlson Comorbidity Index; LPN, laparoscopic partial nephrectomy; LRC, laparoscopic radical cystectomy; LRP, laparoscopic radical prostatectomy; OPN, open partial nephrectomy; ORC, open radical cystectomy; ORP, open radical prostatectomy; PRO, patient reported outcome; RARC, robot-assisted radical cystectomy; RARP, robot-assisted radical prostatectomy; RPN, robot-assisted partial nephrectomy.
